# Ascertaining infectious disease burden through primary care clinic attendance among young Aboriginal children living in four remote communities in Western Australia

**DOI:** 10.1371/journal.pone.0203684

**Published:** 2018-09-17

**Authors:** David Hendrickx, Asha C. Bowen, Julie A. Marsh, Jonathan R. Carapetis, Roz Walker

**Affiliations:** 1 Wesfarmers Centre for Vaccines and Infectious Diseases, Telethon Kids Institute, The University of Western Australia, Perth, Western Australia, Australia; 2 Centre for Child Health Research, University of Western Australia, Perth, Western Australia, Australia; 3 NHMRC Centre for Research Excellence in Aboriginal Health and Wellbeing, Telethon Kids Institute, University of Western Australia, Perth, Western Australia, Australia; 4 School of Medicine, University of Western Australia, Perth, Western Australia, Australia; 5 Department of Infectious Diseases, Perth Children's Hospital, Perth, Western Australia, Australia; 6 Menzies School of Health Research, Charles Darwin University, Darwin, Northern Territory, Australia; Universidade Nova de Lisboa Instituto de Higiene e Medicina Tropical, PORTUGAL

## Abstract

Infectious diseases contribute a substantial burden of ill-health in Australia’s Aboriginal children. Skin infections have been shown to be common in remote Aboriginal communities, particularly in the Northern Territory, Australia. However, primary care data on skin and other infectious diseases among Aboriginal children living in remote areas of Western Australia are limited. We conducted a retrospective review of clinic presentations of all children aged 0 to 5 years presenting to four clinics located in the Western Desert region of Western Australia between 2007 and 2012 to determine this burden at a local level. Infectious diseases accounted for almost 50% of all clinic presentations. Skin infections (sores, scabies and fungal infections) were the largest proportion (16%), with ear infections (15%) and upper respiratory infections (13%) also high. Skin infections remained high in all age groups; 72% of children presented at least once with skin infections. Scabies accounted for only 2% of all presentations, although one-quarter of children presented during the study for management of scabies. Skin sores accounted for 75% of the overall burden of skin infections. Improved public health measures targeting bacterial skin infections are needed to reduce this high burden of skin infections in Western Australia.

## Introduction

The health disparities affecting Indigenous populations around the world are well documented [1]. Indigenous people experience poorer health and social circumstances, which is reflected in a range of key health and wellbeing indicators when compared to non-indigenous or total population benchmarks, including life expectancy at birth, infant mortality, birth weight, child malnutrition and educational attainment [1]. In Australia, the health status of Aboriginal and/or Torres Strait Islander people (hereafter referred to as Aboriginal, for brevity) is marked by a 10 year life expectancy gap [2] and significantly higher rates of chronic [3] and communicable diseases [4] when compared to the non-Aboriginal population. Furthermore, 21.4% of Aboriginal people live in remote and very remote areas of Australia (compared to 1.7% of non-Aboriginal Australians) [5], where life expectancy is lowered further, access to health services is less consistent and the burden of many chronic and communicable diseases is higher compared to Aboriginal people living in urban areas [6–10]. Many socioeconomic, environmental and cultural factors contribute to ill health in rural and remote Australia [11,12], including inadequate housing and overcrowding [13–15], excessive exposure to dust [16] and poor access to health services [17].

In Western Australia (WA), where 40% of the Aboriginal population live in remote or very remote areas [18], the Western Australian Aboriginal Child Health Survey (WAACHS) documented high parent and carer-reported rates of recurring respiratory, ear, skin and gastrointestinal infections in their children between 2000 and 2001, with the highest rates reported among young children living in very remote communities [19]. Self-reported rates of recurring skin infections in children were particularly high in a vast, remote area in the Pilbara region of WA known as the ‘Western Desert’ [19]. This high burden of skin infections among children living in the Western Desert area was also noted in a study on school readiness [20]. Anecdotal reports from teachers, child health nurses and other stakeholders in the Western Desert described high rates of observable infections and illness among children, singling out skin infections as a major concern [20]. The study concluded that the burden of childhood infections was likely to constitute an important barrier to early childhood development, school readiness and engagement and on the subsequent health, economic and social wellbeing of Aboriginal communities in the region [20]. Ensuing community consultations with the regional Aboriginal Medical Service (AMS), school staff and other community members further confirmed the impact of skin infections on child health as a priority health issue. Despite this no recent prevalence or clinic presentation data on skin and other common childhood infections in remote WA Aboriginal communities are available [21].

Here we address this gap and report on the outcomes of a retrospective review of community clinic presentations in the Western Desert. We aimed to ascertain the burden of infectious diseases and health care seeking behaviour in the first five years of life of children living in the Western Desert region. We focussed on scabies and skin sores as exemplar infections, given the high level of concern from community members that had been previously reported [20].

## Methods

### Study setting

The study was conducted in four remote Aboriginal communities located in the Western Desert region of Western Australia, 1,000 to 1,400 km north east of Perth, the state capital. The estimated total population of all four communities is 792, of whom 650 (82%) are Aboriginal people [22]. The communities are located between 150 and 700 km from the nearest regional town and are only accessible by unsealed roads or by small plane (each community has a gravel air strip). In the wet season (from November to April) flooding can make the communities inaccessible. These four communities are culturally linked, all being home to the Martu, a distinct group of Aboriginal people that share a common cultural tradition [23,24].

Each of the four communities has a local clinic, all managed by the same regional AMS. The clinic in the largest community (total population of 427) is usually staffed by two to four nurses and a general practitioner (GP), while the clinics in the three smaller communities (with populations ranging between 81 and 156 people [22]) are generally staffed by a single nurse. All clinic staff are full-time and resident in the community. At the time of data collection, no full-time Aboriginal health workers were based in any of the communities. The GP based in the largest community visits the three smaller communities every few weeks over the course of three consecutive days. External child health services that regularly visit the communities include a specialist paediatrician (approximately every six weeks) and an allied health team (two to three times per year).

### Data collection and analysis

We performed a retrospective review of medical records for all children aged 0 to 5 years that presented to any of the four clinics between the 1^st^ of January 2007 and the 31^st^ of December 2012. Patient consultation records prior to 2010 were only available in hard copy patient files located in each of the clinics, while all patient consultation data from 2010 onwards were accessed through MMEX (ISA Health Care, Perth, WA), a web-based electronic-health platform. We reviewed MMEX consultation records in their entirety, including progress notes and specific diagnosis and treatment fields. We mirrored the data collection process first established by Clucas *et al* [25] and later repeated in two similar studies implemented in remote Aboriginal communities in the Northern Territory (NT) of Australia [26,27].

Eligible children were those registered at clinics in any of the four communities. Frequency of presentations per disease category and per child were summarised by diagnosis either for age group (<1, 1 to <2, 2 to <3, 3 to <4, 4 to <5, 5 to <6 years) or by calendar year (1^st^ of January to 31^st^ of December for each year, 2007–2012). Continuous asymmetric data were expressed as medians with interquartile range (IQR) and dichotomous data as counts and percentages. Individual person-years (time contributed to the study during which the child was in the 0 to 5 year age range) were calculated as the time from birth or 1 January 2007, whichever was later, until the child’s 6^th^ birthday or 31 December 2012, whichever was sooner, or part thereof for age-specific person-years. Children were therefore followed for variable lengths of time. It was assumed that all children registered at one of the four remote community clinics were resident in the community from birth and remained in the community until their sixth birthday, therefore, the person-years (rate denominators) are potentially over-estimated since the frequencies of presentations (rate nominator) are potentially under-estimated due to birth elsewhere or movement to other locations. Because these are relatively stable communities with limited population movement, the age- and year-specific rates were thought to be reasonable approximations. Age- and year-specific rates were also calculated using 2011 Australian census data for these communities [22]. All 95% confidence intervals were calculated using Poisson exact methods. Data were analysed in R version 12.1 (R Project for Statistical Computing, City, State http://www.R-project.org). Administered or prescribed treatment data are reported for new skin sore presentations only (referred to here as ‘standard’ presentations) and exclude any possible follow-up visits (which were coded as ‘review’ presentations). This was done to capture only new treatment prescriptions for skin infections and to avoid overrepresentation of oral antibiotics in our results; BPG is administered as a single dose in one clinic presentation, while oral antibiotic courses are usually 7 to 10 days and often required several ‘review’ presentations to complete the course.

Date of birth, gender and community of residence were recorded. For each episode we recorded: date of presentation, the child’s height and weight when documented, the reason(s) for presentation, prescribed treatment and whether the child was referred to hospital for treatment or a specialist consultation. The reasons for presentation were categorised *a priori* and according to the classification described in Clucas *et al* [25]. Our data collection form recorded the following reasons for presentation (multiple reasons per presentation possible): scabies, skin sores, acropustulosis (added in after community consultation determined this might be a frequent diagnosis), dermatophytes, eye infections (with a separate indicator for trachoma), ear infections (any of acute otitis media, otitis externa, chronic suppurative otitis media), throat infections, post streptococcal glomerulonephritis, acute rheumatic fever, lower respiratory tract infection, upper respiratory tract infection, asthma, diarrhoea and other febrile illness (fever recorded and no other reason for presentation documented). We classified as ‘non-infectious’ any presentations for one or more of the following: routine and scheduled child health checks without infectious diagnosis, specialist and allied health appointments for non-infectious causes, immunisations, fractures and minor trauma, and any other symptomatic presentations for which no infectious cause was identified. Repeat presentations for the same disease category within a 2-week period were considered as the same episode of disease and were coded as ‘review’ presentations, unless clinic consultation notes stated otherwise. We also documented hospital referrals, which included urgent evacuations (including retrievals by the Royal Flying Doctor Service), and referrals to a regional hospital for tests. The patient record review and data-entry was performed by DH from October 2013 to November 2015.

Ethics approval was obtained from the Western Australian Aboriginal Health Ethics Committee and the University of Western Australia Human Research Ethics Office (references 477 and RA/4/1/6563). Waiver of individual consent was provided. The study was discussed with the regional AMS and their input was sought in preparation of the study.

## Results

There were a total of 304 children under the age of 6 years with available data. This total decreased to 231 (76.0%) children (61% male) after removing records for children whose patient files indicated a place of residence other than the four communities that were the focus of our study. These 4 clinics provided care on at least one occasion for 73 children resident elsewhere (24.0% of all presenting children), suggesting a high rate of community mobility in the wider region. There were 7,504 individual clinic presentations for the 231 children, equating to on average 32.5 visits / child or 10.4 /person-year (7,504 presentations/724.1 person-years). In all age groups, > 80% of children visited the clinic in a calendar year. This was highest in infancy (<1year) and the oldest age group (5 –<6 year olds). Data were collected from birth for 116 children. Approximately 100 children contributed an entire calendar-year in each age group and about 120 person-years were contributed to each age group and calendar year rate estimate (Table 1).

**Table 1:** Study participants: Children under 6 years registered at Western Desert community health clinics, January 2007 to December 2012.

age in years	population estimate (census 2011)	# children registered at the clinics	# children with data for entire year		% of children with at least 1 presentation^a^	person-years	median (IQR) number of presentations	
							all children^b^	presenting children^c^
< 1	90	136	100	(73.5%)	87.0%	117.8	13.5 (4.8,22.0)	17.0 (10.0,23.5)
1 to <2	96	139	104	(74.8%)	82.7%	119	13.0 (3.0,21.0)	16.0 (8.3,22.8)
2 to <3	54	143	98	(68.5%)	79.6%	120.7	10.0 (1.3,16.0)	12.0 (7.0,17.0)
3 to <4	156	141	102	(72.3%)	82.4%	120.7	6.0 (2.0,11.8)	9.0 (4.8,12.0)
4 to <5	66	145	100	(87.0%)	85.0%	123.8	6.0 (2.0,12.0)	7.0 (4.0,13.0)
5 to <6	72	147	95	(64.6%)	89.5%	122.1	6.0 (3.0,11.0)	7.0 (4.0,11.0)

#, number; %, percentage; IQR, interquartile range

^**a**^ Proportion denominator is # children registered at community clinics included in the study, for children in the specific age group, over the entire study period.

^**b**^ Presentations among all eligible children for whom data is available for entire year (includes children with no presentations).

^**c**^ Presentations among children who have presented at least once for the age group (excludes children with no presentations).

**Fig 1:**
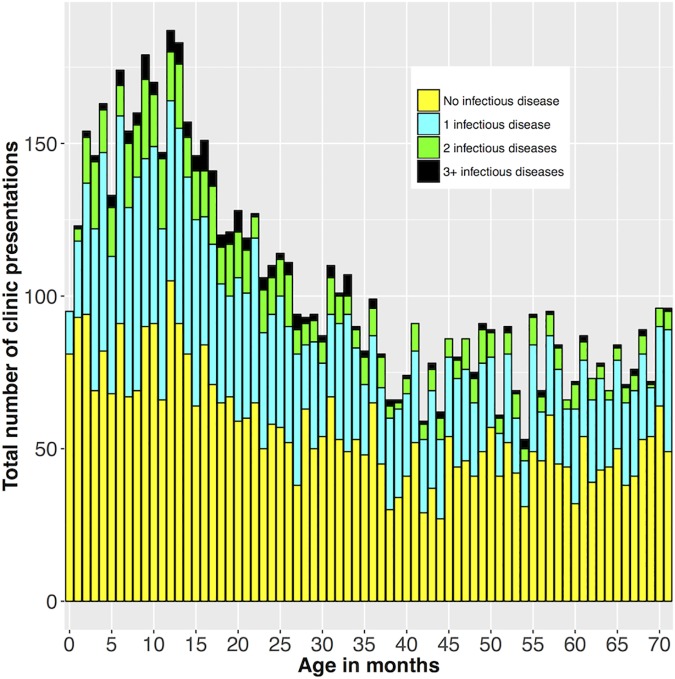
Age-specific frequency of clinic presentations for children under 6 years by number of infectious disease diagnoses at presentation.

Approximately half of all recorded clinic presentations were for infectious disease diagnoses (Table 2 and Fig 1), a proportion that was maintained across all ages. Ear infections, upper respiratory tract infections and skin sores were the most frequently diagnosed infections, accounting for 15%, 13% and 12% respectively. These infection types were all experienced by the majority of children at least once in the time frame of our study (66%, 75% and 72% of all included children, respectively).

**Table 2:** Reasons for presentation of children under 6 years of age at four Western Desert community health clinics, January 2007 to December 2012.

Reason for presentation	Number (%) of presentations^a^ N = 7504		Number (%) of children presenting^a^ N = 231	
**non-infectious presentation**^**a**^	4088	(54%)	225	(97%)
**respiratory infections**				
upper RTI	966	(13%)	173	(75%)
lower RTI	414	(6%)	118	(51%)
**skin infections**				
skin sores	935	(12%)	167	(72%)
fungal	148	(2%)	60	(26%)
scabies	137	(2%)	60	(26%)
crusted scabies	6	(<0.1%)	4	(2%)
acropustulosis	11	(0.1%)	4	(2%)
**other infections**				
ear infections	1138	(15%)	152	(66%)
throat infections	214	(3%)	104	(45%)
other febrile illness	127	(2%)	77	(33%)
**complications associated with GAS**				
APSGN	8	(0.1%)	2	(1%)
acute rheumatic fever	1	(<0.1%)	1	(<1%)
rheumatic heart disease	1	(<0.1%)	1	(<1%)
**other**				
diarrhoea	534	(7%)	135	(58%)
asthma	6	(<0.1%)	6	(3%)
hospital referrals	61	(1%)	45	(19%)

%, percentage; RTI, respiratory tract infection; GAS, group A streptococcus; APSGN, acute post-streptococcal glomerulonephritis.

^a^ More than one reason can be recorded at each presentation

The number of clinic presentations per child per age category (Table 1) was highest in the first two years of life, with a median of around 13 presentations per year, and steadily declined to a median of 6 presentations per year by school age, although there was considerable variability in the individual rates. Overall, 33.8% of clinic presentations involved one infection, 9.3% two infections and 2.4% involved three or more infections. These proportions were constant across all age groups (Fig 1). One in five children were referred to a hospital for treatment or a specialist consultation over the course of this study.

Skin sore presentations affected all age groups with almost three quarters of children presenting at least once with skin sores. The highest presentation rate for skin infections was in 5 –<6 year olds (162/100 person years; 95% confidence interval [CI] 140–186). Whilst presentation to the clinic was high, treatment administered was variable and inconsistent with the recommended treatment guidelines in place at the time of this audit, namely the manual of the Central Australian Rural Practitioners Association (CARPA) which recommends all children with skin sores receive a single dose of intramuscular benzathine penicillin G (BPG) [28]. A dose of BPG alone was administered in 30 (5%) of a total of 563 standard presentations for skin sores, while oral antibiotics alone and topical antibiotics alone were prescribed in 191 (34%) and 79 (14%) of standard skin sore presentations respectively. Combined oral and topical antibiotics were prescribed in 90 (16%) of standard skin sore presentations. Contrary to guidelines, no antibiotics were prescribed in 158 (28%) of standard skin sore presentations.

**Fig 2:**
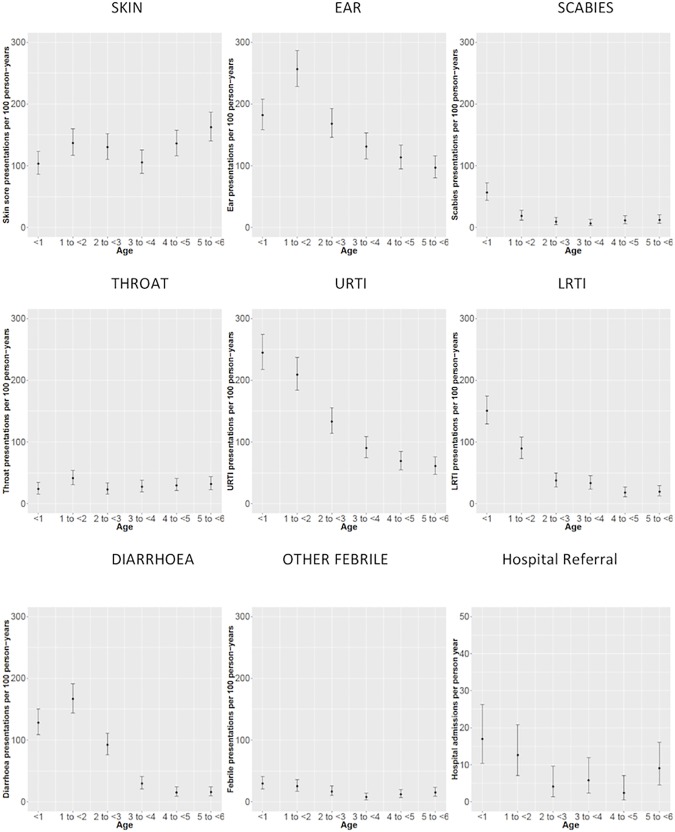
Rates of disease-specific presentations, by age, for children under 6 years of age at four Western Desert community health clinics, January 2007 to December 2012.

Overall, 2% (n = 137) of all presentations were for children presenting with scabies, 51 (37.2%) of which also included a diagnosis of skin sores. One quarter of children presented at least once for a scabies infestation. The rate of scabies presentations was highest in infants (57/100 person years; 95% CI 44–72). One quarter of all scabies presentations occurred before the age of 6 months and scabies had the youngest median age of onset for all infections suggesting very early acquisition of this parasitic disease. (Table 3 and Fig 2) Scabies was less likely to be recognised in older children, whilst skin sores remained common.

**Table 3:** Median age at first clinic presentation for scabies, skin sores, ear infections, URTIs and LRTIs, January to December 2012.

Reason for presentation	n	Median age in years at first presentation (IQR)
Scabies	137	1.11 (0.46,3.08)
Skin sores	935	3.23 (1.79,4.82)
Ear infections	1138	2.17 (1.19,3.88)
LRTI	966	1.68 (0.80,3.23)
URTI	414	1.18 (0.58,2.54)

IQR, interquartile range; LRTI, lower respiratory tract infection; URTI, upper respiratory tract infection

**Fig 3:**
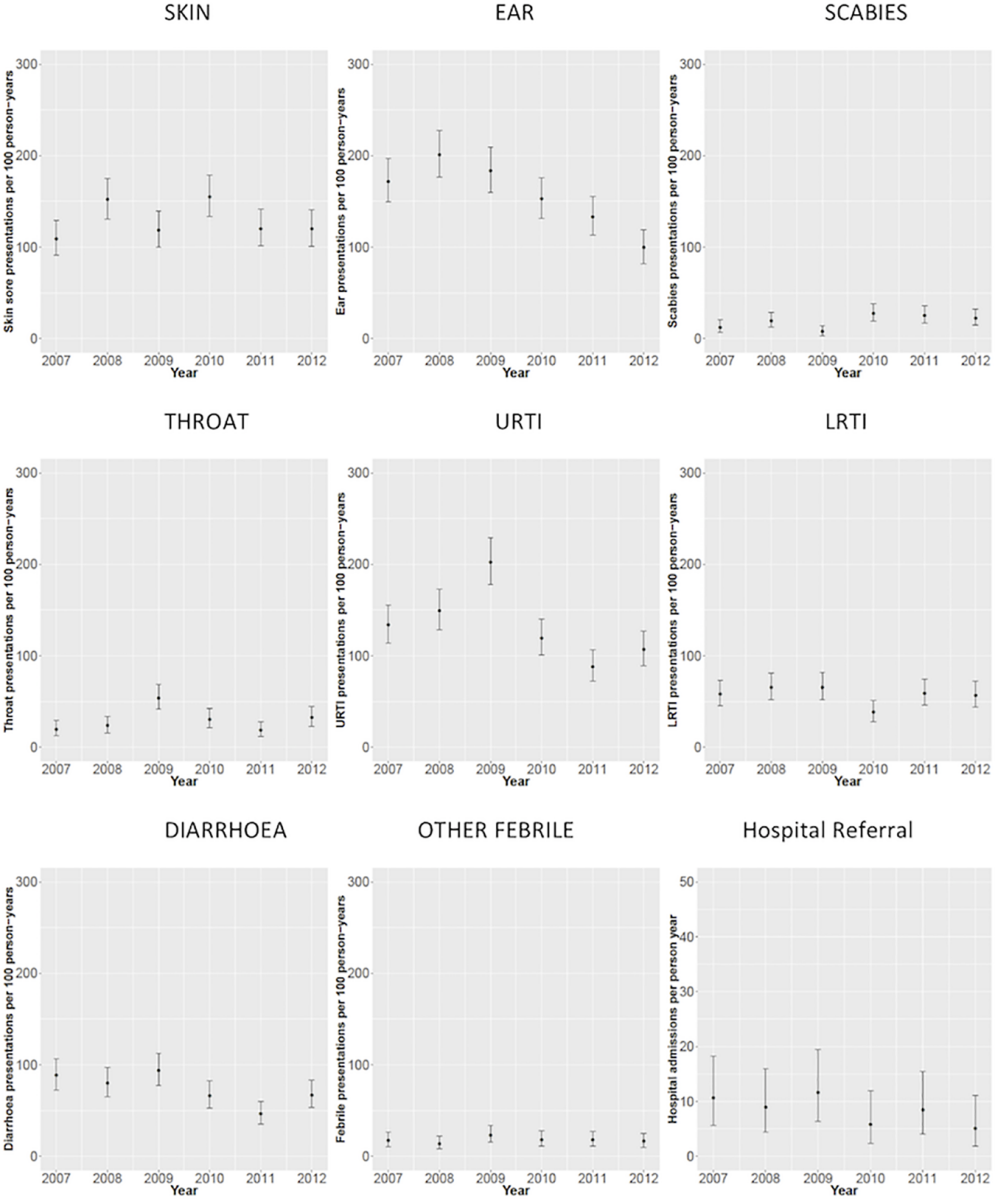
Rates of disease-specific presentations, by year, for children under 6 years of age at four Western Desert community health clinics, January 2007 to December 2012.

Infectious disease associated clinic presentation rates were relatively constant between 2007 and 2012 (Fig 3), with the exception of presentations for URTIs and ear infections, which showed some evidence of a reduction over time, especially for the latter. However, there was large variability in the disease-specific presentation rates across the age groups (Fig 2). Presentation rates for ear infections, upper respiratory tract infections (URTIs), lower respiratory tract infections (LRTIs) and gastrointestinal infections were higher in children up to the age of 3 years when compared to older children.

## Discussion

We report a high burden of primary health care presentations, particularly in the first three years of life, amongst Aboriginal children living in remote communities in the Western Desert area of the Pilbara region, WA. Although some of these presentations would be for routine child health checks and immunisations, almost half of all child presentations were for infectious causes, amongst which skin infections (sores, scabies and fungal infections combined) were the largest proportion (16%), with ear infections (15%) and upper respiratory infections (13%) also high. Skin sore presentations remained high throughout all age groups, with a slight peak observed in the older age group (5-<6 year olds), whilst presentations for ear infections and URTIs declined with age. Our findings confirm the anecdotal reports of a high burden of infectious diseases in the Western Desert area [19,20], dominated by skin infections across all age groups [19,20,29,30]. Infectious presentations remained high throughout the study period, with a slight decline only seen in ear infection presentations over time. Whilst it is encouraging to see a downwards trend in the high burden of ear infections, there is a pressing need to evaluate current prevention and control strategies for common childhood infections in the region.

Overall, our study shows that at least one child aged 0–5 years presents to a Western Desert community clinic every single day, and that every second day this would be for an infectious cause. This age group is well recognised as having high health needs [31,32]. Our findings show this is also true in the remote Aboriginal context and indicates that overall parents are utilising community health services in the Western Desert and bringing their children to the clinic. We found that health service utilisation was particularly high for infants, as has also been shown to be the case in other remote Aboriginal community settings [33]. However, we report a lower median number of primary care presentations for infants and young children compared to those documented in other remote Aboriginal community settings [25–27,33]. This could be due to an actual lower burden of disease, but could also be a reflection of variability in health service utilisation practices.

Akin to the findings of similar community clinic audit studies undertaken in the NT [25–27], we found that the high number of community clinic presentations amongst children for infectious causes is driven primarily by skin, respiratory and ear infections. Hence health care practitioners need to be well positioned to diagnose and treat these conditions in children living in remote Aboriginal communities. Diagnosis and clinical management resources such as the CARPA manual [34] are intended to support primary care providers operating in remote Aboriginal communities by providing standardised, evidence-based guidelines for their practice. However, in our assessment of skin sore treatment we found that while the CARPA manual was available in the community clinics, treatment guidelines were often not strictly adhered to. While a single intramuscular dose of benzathine penicillin was recommended as the first line of treatment for skin sores at the time of the study [34], we found this was only administered in 5% of standard skin sore presentations. Oral antibiotic courses were prescribed in most cases, notwithstanding concerns about treatment adherence [35,36]. Furthermore, we found that a topical antibiotic alone (mupirocin) was prescribed in one out of every seven standard skin sore presentations, when the CARPA manual advises against its use out of concern of increasing drug resistance [34]. These findings suggest that health care practitioners do not necessarily follow current skin infection treatment guidelines and that other factors affect their clinical management practices, as has been documented previously for other maternal and child health issues in remote Aboriginal communities [37]. We have performed a qualitative study to better understand clinical management practices and other community aspects of skin infections in the Western Desert, the results of which will be reported elsewhere.

The consistently high rate of skin sores we reported throughout our study period and across all age groups is concerning considering the acute and chronic sequelae that are associated with bacterial skin infections. Prolonged exposure to the causative bacteria of these infections (*Staphylococcus aureus* and *Streptococcus pyogenes*) can lead to a range of complications, including invasive disease [38]. Indeed, the high prevalence of group A streptococci in Australia’s remote communities is the key determinant of the excessive burden of glomerulonephritis, acute rheumatic fever and rheumatic heart disease in Australia’s Aboriginal population [39–41]. Early and effective treatment of skin infections at the primary care level is therefore crucial. However, high skin infection associated hospitalisation rates amongst Aboriginal children in WA, particularly amongst children living in remote areas, indicate that substantial improvements to skin infection primary care in remote Aboriginal communities are urgently required [8,42].

While we reported high community clinic presentation rates for scabies in infants, our data suggest a substantially lower overall burden for scabies in the Western Desert compared to remote communities in the NT [25–27]. Given that scabies is considered an important contributor to bacterial skin sores in remote Aboriginal community settings [26,27,38], it is of note that we reported similarly high numbers of skin sore presentations in the Western Desert. This suggests that even in settings with relatively low rates of observed scabies, structural factors such as overcrowding, housing conditions, minor trauma and insect bites driven by inadequate environmental health can still drive the incidence of skin sores in children living in remote Aboriginal communities to extreme levels [13–16]. Alternatively, this might also be a reflection of the long history of research and public health campaigns on scabies in the NT, which possibly leads to an increased awareness and ability to diagnose scabies amongst health services operating there. Scabies might therefore be underdiagnosed in rural and remote WA, as shown in a recent study [43].

Recurring infections affect school readiness, school attendance and physical development of Aboriginal children in remote communities [13,20,44–46]. A recent study showed that severe childhood infection requiring hospital admission is associated with developmental vulnerabilities [47], which suggests that the developmental deficits previously documented among Aboriginal children living in the Western Desert [20,48] are likely to be at least in part due to the high burden of childhood infections found in these communities. Our findings further underscore the importance of addressing this high burden of infections, and particularly those of the skin, in remote Aboriginal communities; reducing the burden of infectious diseases is likely to benefit overall child development.

Our study was based on a retrospective review of community clinic records, both in the form of hand-written notes (for consultations from 2007 to 2010) and a digital format (for consultations from 2010 onwards). This type of study is largely dependent on the quality of these consultation notes. Since diagnosis in these settings is typically based on clinical signs and symptoms, it relies greatly on the experience and knowledge of the healthcare provider, in addition to the use of standardised clinical guidelines such as the CARPA manual [34]. Laboratory confirmation for the infections discussed here is rare. Misdiagnosis might therefore occur, and at times variability between health providers in diagnosing cases was apparent in the consultation notes. Under-diagnosis of skin infections might also occur [43]. Given the study design, its time frame and the age range we focussed on (0 to 5 years), children were followed for variable lengths of time. One additional limitation of this study is that due to logistical limitations we were unable to perform a systematic validation of the quality of the entered data beyond logic checks. However, we used a standardised data collection protocol [25] to minimise the chance of systematic data entry error. Furthermore, the rates reported here are potentially subject to underreporting of events and overestimation of the person years since the exact movement of the children is unknown during the study period. Replacement of person-years in these calculations with Australian census data would not correct for the over-estimation of individuals at risk since these official statistics may under-represent our study population [49].

We conclude that infectious diseases are an important reason for Aboriginal children to present to community clinics in the Western Desert. The burden of skin, respiratory and ear infections are particularly high. Notwithstanding a reduction of presentation rates for some infections over time (URTI, ear infections), skin sore presentation rates remained high over time and across all ages. The high rates of primary care presentations due to infectious causes in remote Aboriginal communities documented in this and other studies [21] indicates a crucial need for improved prevention and control strategies. The high burden of skin infections, the severe sequelae that can arise, and the availability of practical, community-based control strategies to reduce this burden justify an increased focus on this important public health issue in remote WA.
